# Chronic inflammation aggravates metabolic disorders of hepatic fatty acids in high-fat diet-induced obese mice

**DOI:** 10.1038/srep10222

**Published:** 2015-05-14

**Authors:** Lei Zhao, Shan Zhong, Haiyang Qu, Yunxia Xie, Zhennan Cao, Qing Li, Ping Yang, Zac Varghese, John F. Moorhead, Yaxi Chen, Xiong Z. Ruan

**Affiliations:** 1 Centre for Lipid Research, & Key Laboratory of Molecular Biology on Infectious Diseases, Ministry of Education, the Second Affiliated Hospital, Chongqing Medical University, Chongqing, China; 2 John Moorhead Research Laboratory, Centre for Nephrology, University College London Medical School, Royal Free Campus, University College London, London, UK

## Abstract

The prevalence of nonalcoholic fatty liver disease (NAFLD) increases with increasing body mass index (BMI). However, approximately 40–50% of obese adults do not develop hepatic steatosis. The level of inflammatory biomarkers is higher in obese subjects with NAFLD compared to BMI-matched subjects without hepatic steatosis. We used a casein injection in high-fat diet (HFD)-fed C57BL/6J mice to induce inflammatory stress. Although mice on a HFD exhibited apparent phenotypes of obesity and hyperlipidemia regardless of exposure to casein injection, only the HFD+Casein mice showed increased hepatic vacuolar degeneration accompanied with elevated inflammatory cytokines in the liver and serum, compared to mice on a normal chow diet. The expression of genes related to hepatic fatty acid synthesis and oxidation were upregulated in the HFD-only mice. The casein injection further increased baseline levels of lipogenic genes and decreased the levels of oxidative genes in HFD-only mice. Inflammatory stress induced both oxidative stress and endoplasmic reticulum stress in HFD-fed mice livers. We conclude that chronic inflammation precedes hepatic steatosis by disrupting the balance between fatty acid synthesis and oxidation in the livers of HFD-fed obese mice. This mechanism may operate in obese individuals with chronic inflammation, thus making them more prone to NAFLD.

Obesity is an independent risk factor for nonalcoholic fatty liver disease (NAFLD), which is characterized by increased intrahepatic triglyceride (TG) content (steatosis) with or without inflammation and fibrosis (i.e., steatohepatitis). Interestingly, approximately 40–50% of obese adults do not develop hepatic steatosis^1^,^2^, raising the question as to why certain obese people are more prone to developing NAFLD. Unraveling the mechanisms that link obesity to hepatic steatosis requires complex interdisciplinary research.

Obesity and NAFLD are independently associated with chronic inflammation, which is characterized by abnormal cytokine production, increased acute-phase reactants and other mediators as well as activation of a network of inflammatory signaling pathways^3^. Moreover, obese patients with hepatic steatosis and steatohepatitis present with higher levels of C-reactive protein (CRP) and other inflammatory biomarkers compared with age-, sex- and obesity-matched controls^4^,^5^. The production of cytokines (e.g., interleukin-6, IL-6 and tumor necrosis factor-α, TNFα) and chemokines (e.g., monocyte chemoattractant protein 1, MCP-1) in adipose tissue is greater in obese subjects with NAFLD than body mass index (BMI)-matched subjects with normal intrahepatic TG content^6^. Despite the fact that numerous studies have reported important and complex interactions between chronic inflammation, obesity and hepatic steatosis^7^, it remains unclear whether inflammation initiates the intrahepatic biochemical changes observed in steatotic livers.

Casein is a major component of cow milk proteins and can stimulate B-lymphocytes and trigger a non-infectious systemic inflammatory response^8^. Endotoxin (lipopolysaccharide, LPS), a key constituent of many bacteria, plays a central role in the innate immune response and has been used in previous NASH models^9^. However, LPS-induced inflammation has the potential to cause endotoxic septic shock, a condition that often leads to multiple organ failure and mortality^10^. Compared with LPS, cytokine-only-treated animal models or local inflammation models, the inflammation induced by subcutaneous injection of casein is characterized by increases in the levels of multiple cytokine/chemokines in the serum as well as increased serum amyloid A (SAA, which is like human CRP, a well-known marker for the systemic inflammatory response in patients)^11^. Therefore, the casein injection model is more likely to mimic the chronic systemic inflammatory state observed in patients. In addition, it has been widely used in animal research on atherosclerosis, amyloidosis, chronic kidney disease and liver disease^12^,^13^,^14^.

In the present study, we subjected C57BL/6J mice to a high-fat diet (HFD) in the absence or presence of a casein injection for 14 weeks. Although mice on a HFD exhibited apparent phenotypes of obesity and hyperlipidemia regardless of exposure to casein injection, only the HFD+Casein mice showed the typical characteristics of vacuolar degeneration and inflammation in the liver compared with mice on a normal chow diet (NCD, control). Compared to previous studies, our work dissociates inflammation from obesity. Our model allows us to investigate the exact role of inflammation in the progression of NAFLD and to explore the underlying mechanisms.

## Results

### Casein injection causes chronic inflammation in HFD-induced obese mice

**Figure 1:**
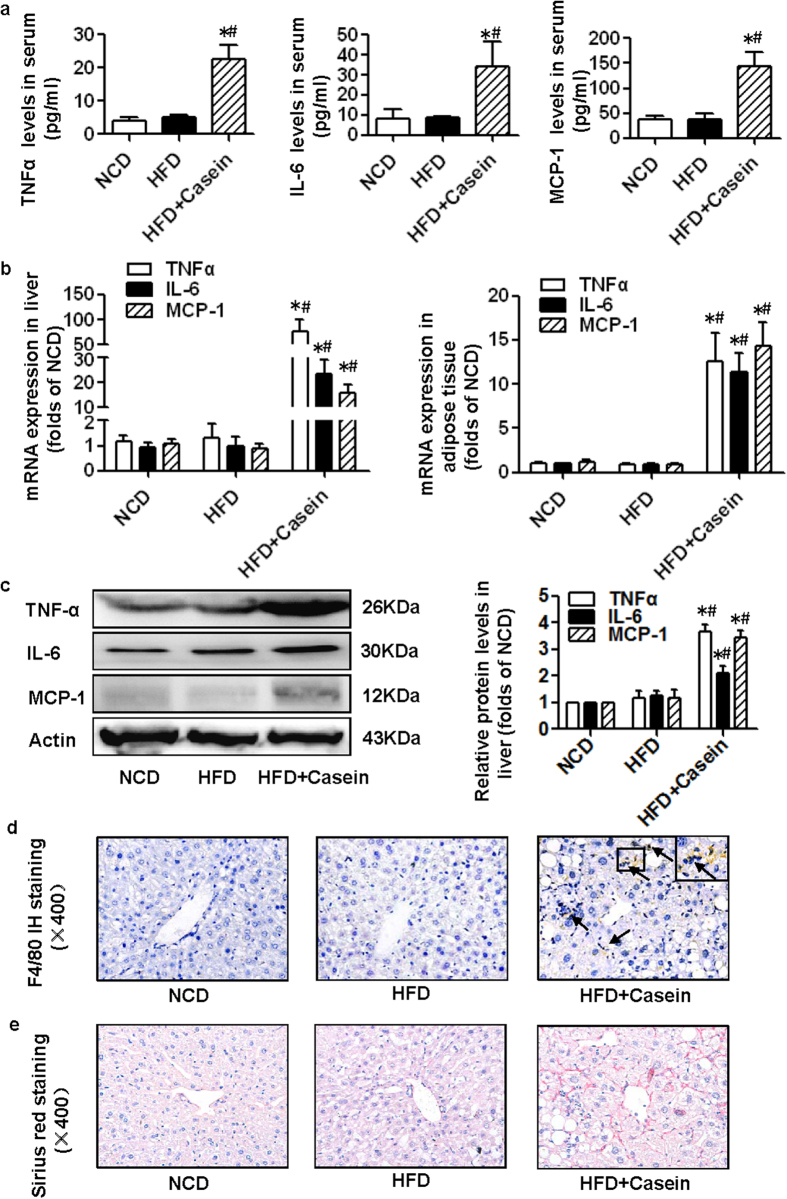
Casein injection induced chronic systemic inflammation in C57BL/6J mice. Mice were fed a normal chow diet (NCD), high-fat diet (HFD) or HFD plus casein injection (HFD+Casein) for 14 weeks. (**a**) The levels of TNFα, IL-6 and MCP-1 in the serum of C57BL/6J mice were assessed using a Milliplex ELISA assay kit (*n* = 6). (**b**) The mRNA expression of TNFα, IL-6 and MCP-1 in the livers and adipose tissues of C57BL/6J mice (*n* = 8). (**c**) The protein levels of TNFα, IL-6 and MCP-1 in the livers of C57BL/6J mice (*n* = 3). Results are depicted as the mean ± SE, *P < 0.05 versus NCD, #*P* < 0.05 versus HFD. (**d**) F4/80-positive macrophages (brown) in the livers of C57BL/6J mice. (**e**) Histopathological examination of mouse livers by Sirius red staining. Representative photomicrographs of liver sections from NCD, HFD and HFD+Casein mice are shown.

TNFα, IL-6 and MCP-1 serum levels were unchanged in the HFD group; however, these cytokine/chemokines were significantly elevated in the serum of HFD+Casein mice compared with control mice (Fig. 1a). We also examined inflammatory cytokines produced in the liver and adipose tissues. The expression of IL-6, TNFα and MCP-1 was not significantly increased in these two tissues in the HFD group, but the combined presence of HFD and casein injection induced significantly elevated cytokine levels compared with controls or the HFD group (Fig. 1b, c). Immunohistochemical analysis of hepatic sections revealed that the number of F4/80-positive macrophages was markedly increased in the livers of HFD+Casein mice (Fig. 1d). The liver fibrosis in the HFD+Casein group was also confirmed by Sirius red staining (Fig. 1e), and the quantitative data in HFD+Casein group was 13.32% ± 0.74% Sirius-red positive area per field vs. 4.03% ± 0.19% in controls and 4.48% ± 0.32% in HFD group. These data suggest that casein injections successfully induced chronic systemic inflammation in HFD-fed mice.

### Metabolic profile of control, HFD and HFD+Casein mice

The body weight, body weight increase, daily caloric intake, epididymal fat weight, serum TG and serum free fatty acid (FFA) levels were significantly higher in HFD and HFD+Casein mice compared with controls (Table 1). These parameters were not significantly different between HFD and HFD+Casein mice, indicating that obesity and hyperlipidemia were successfully induced by HFD feeding and that the casein injection had no further effect on these aforementioned metabolic parameters. However, liver weights were clearly increased in the HFD+Casein group. The fasting glucose concentrations in the HFD+Casein mice were also elevated compared with HFD or control mice, which is possibly due to impaired islet function, as we have described previously^15^.

### Chronic inflammation promotes the development of NAFLD in HFD-induced obese mice

**Figure 2:**
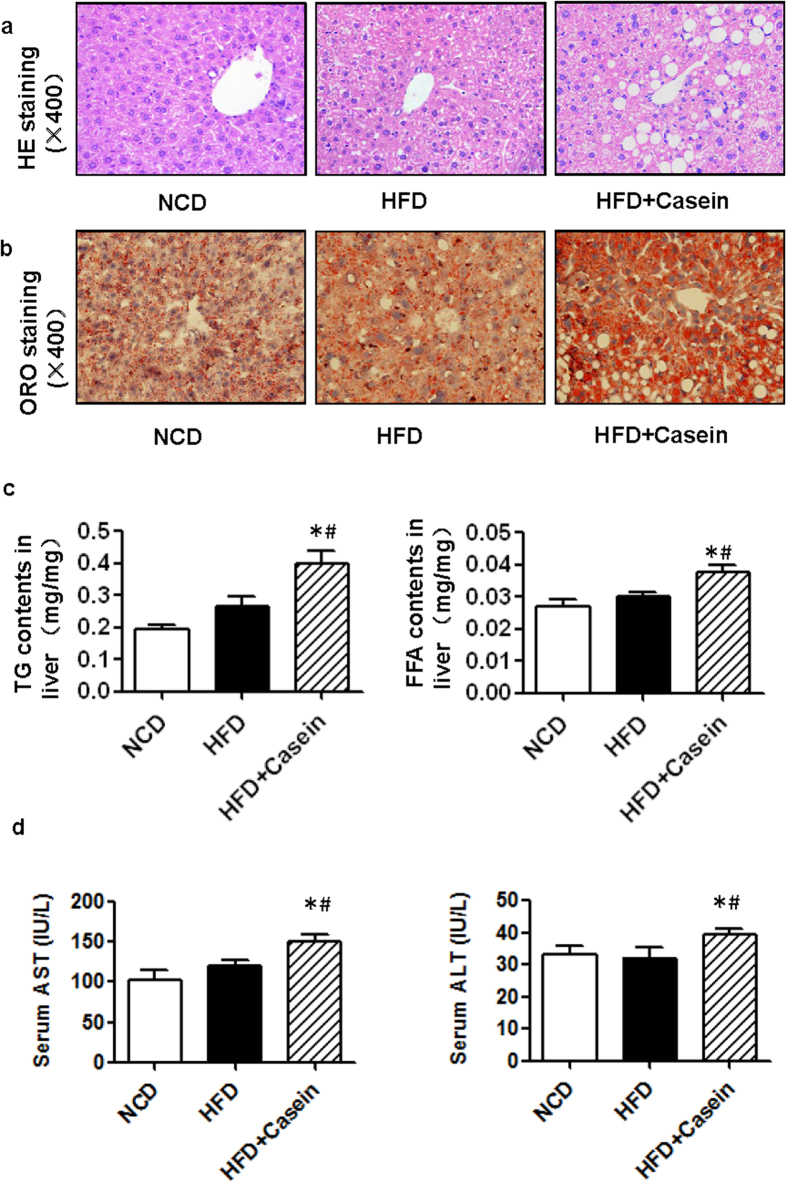
Effect of inflammatory stress on lipid accumulation in the livers of C57BL/6J mice. Mice were fed a normal chow diet (NCD), high-fat diet (HFD) or HFD plus casein injection (HFD+Casein) for 14 weeks. The liver of each mouse was perfused with PBS, embedded and cut as described in Materials and Methods (n = 8). Each section was stained with H&E (**a**) or Oil Red O (**b**). The area indicated by the arrow shows infiltrated inflammatory cells. Representative photomicrographs of the liver sections from NCD, HFD and HFD+Casein mice are shown. (**c**) The concentrations of liver TGs and FFAs. (**d**) The levels of AST and ALT in the serum. The results are depicted as the mean ± SE (*n* = 8), *P < 0.05 versus NCD, #*P* < 0.05 versus HFD.

Next, we performed a histological analysis of the liver (Fig. 2). Hematoxylin and eosin (H&E) staining revealed no apparent vacuolar degeneration in the HFD group compared with controls, while marked vacuolar degeneration was observed in HFD+Casein mice, indicating fat accumulation in the liver (Fig. 2a). Oil Red O staining showed that the number of lipid droplets clearly increased in HFD+Casein mice (Fig. 2b). The quantitative measurements of intrahepatic TG and FFA levels also showed no significant increase in the HFD group; these concentrations were significantly increased in the HFD+Casein group compared with controls (Fig. 2c). In addition, only the HFD+Casein mice had a significant increase in the enzymes aspartate transaminase (AST) and alanine transaminase (ALT) in the serum, indicating impaired liver function (Fig. 2d). These findings are consistent with the histological evaluations of the livers from these three groups.

### Chronic inflammation disrupts the balance between *de novo* fatty acid synthesis and β-oxidation in the livers of HFD-induced obese mice

Because hepatic *de novo* fatty acid synthesis and β-oxidation both contribute to excess lipid accumulation in the liver, we examined several key transcription factors and enzymes that control these two metabolic pathways. Sterol regulatory element-binding protein-1 (SREBP-1) is a nuclear transcription factor that transcriptionally activates nearly all the genes involved in *de novo* lipogenesis^16^,^17^. Acetyl-CoA carboxylase (ACC) and fatty acid synthase (FAS) are important downstream target genes of SREBP-1 and are the rate-limiting enzymes of palmitate formation. Moreover, carnitine palmitoyltransferase 1 A (CPT1A), hydroxyacyl-CoA dehydrogenase beta subunit (HADHB) and carnitine O-octanoyltransferase (CROT), key enzymes involved in intrahepatocellular fatty acid oxidation, mediate the transport of long-chain fatty acids into the mitochondrial matrix, catalyze mitochondrial β-oxidation to liberate carbon units and catabolize very long-chain fatty acids (>20 carbons), respectively^18^,^19^,^20^.

**Figure 3:**
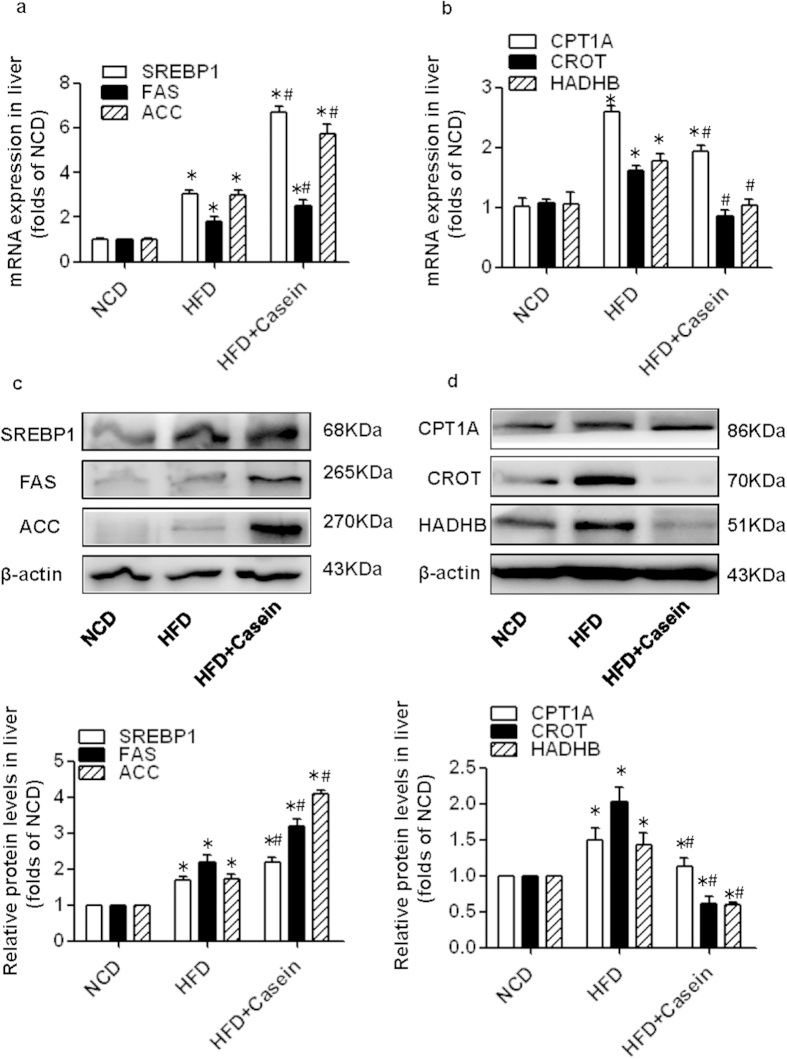
Effect of inflammatory stress on the mRNA and protein expression of fatty acid lipogenic and oxidative genes in the livers of C57BL/6J mice. Mice were fed a normal chow diet (NCD), high-fat diet (HFD) or HFD plus casein injection (HFD + Casein) for 14 weeks. (**a**) The mRNA expression of genes associated with *de novo* fatty acid synthesis. (**b**) The mRNA expression of genes associated with fatty acid oxidation. The results are depicted as the mean ± SE (*n* = 8). (**c**) The protein expression of genes associated with *de novo* fatty acid synthesis. (**d**) The protein expression of genes associated with fatty acid oxidation. The histogram represents the mean ± SE of the densitometric scans for target protein bands from four western blot experiments normalized by comparison to β-actin and expressed as fold change relative to the control (*n* = 4). *P < 0.05 versus NCD, #*P* < 0.05 versus HFD.

Compared with controls, HFD feeding alone significantly upregulated the hepatic mRNA expression of SREBP1, FAS, and ACC by approximately 2-fold, 1-fold and 2-fold, respectively, and were further elevated to approximately 7-fold, 3-fold and 6-fold in HFD+Casein mice, respectively (Fig. 3a). In contrast, the mRNA expression levels of CPT1A, CROT and HADHB were increased by approximately 2-fold, 1-fold and 1-fold in the livers of HFD-fed mice, but the expression levels of these transcripts were decreased to approximately 0.7, 0.5 and 0.6 of HFD values in the HFD+Casein group, respectively (Fig. 3b). Moreover, the protein levels of lipogenic (SREBP1, FAS and ACC) and oxidative genes (CPT1A, CROT and HADHB) were increased in the HFD group. Casein injection further increased the baseline protein expression levels of these lipogenic genes and decreased the baseline protein levels of oxidative genes in the HFD group. Our data indicate that HFD feeding alone could induce a compensatory increase in hepatic fatty acid synthesis and oxidation in an effort to maintain metabolic homeostasis; however, casein injection disrupted the balance between fatty acid production and consumption in the livers of HFD-fed mice, leading to the development the hepatic steatosis.

**Figure 4:**
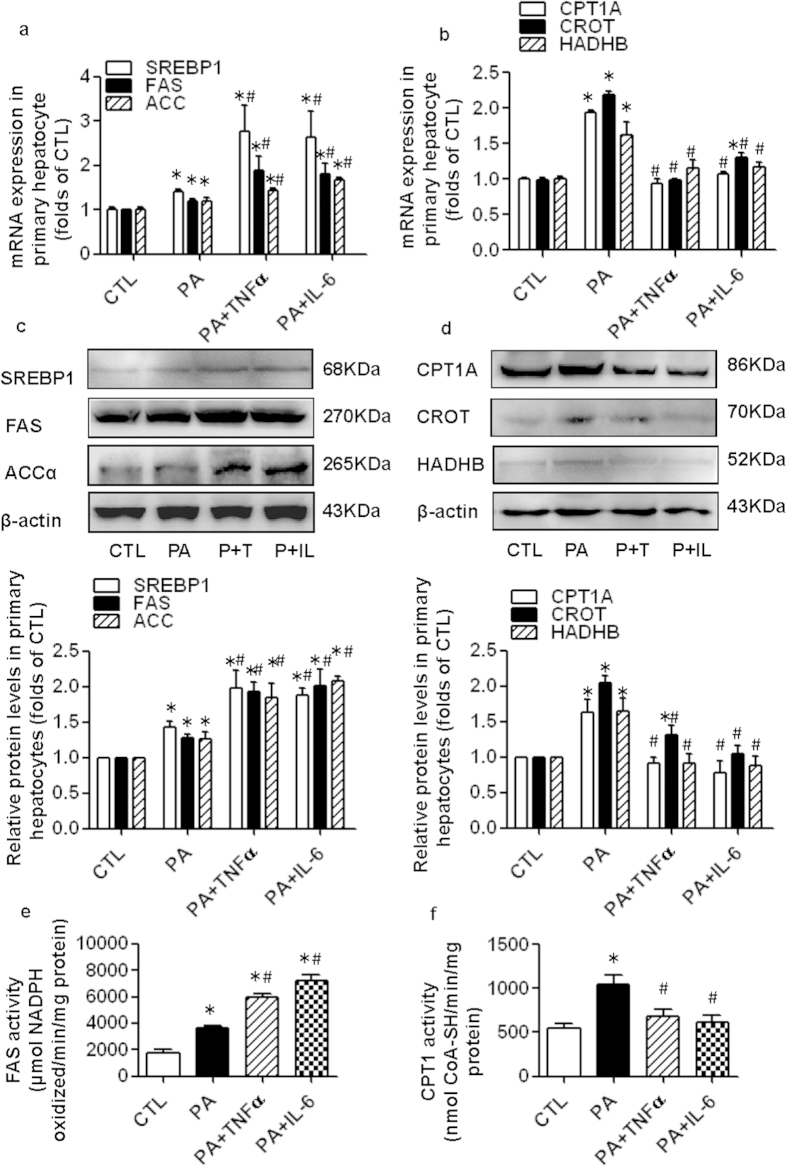
Effect of inflammatory cytokines on the expression and enzymatic activity of fatty acid lipogenic and oxidative genes in primary mouse hepatocytes. Cells were incubated in serum-free medium (control, CTL) or medium containing 0.04 mM palmitate (PA) or 25 ng/ml TNFα plus 0.04 mM palmitate (PA + TNFα) or 20 ng/ml IL-6 plus 0.04 mM palmitate (PA + IL-6). (**a**) The mRNA expression of genes associated with *de novo* fatty acid synthesis. (**b**) The mRNA expression of genes associated with fatty acid oxidation. RNA was extracted, and gene expression was determined by real-time PCR. The results are depicted as the mean ± SE (*n* = 6). (**c**) The protein expression of genes associated with *de novo* fatty acid synthesis. (**d**) The protein expression of genes associated with fatty acid oxidation. The histogram represents the mean ± SE of the densitometric scans for target protein bands from three western blot experiments. (**e**) The enzymatic activity of FAS. (**f**) The enzymatic activity of CPT1. The results are depicted as the mean ± SE (*n* = 4). *P < 0.05 versus CTL, #*P* < 0.05 versus PA.

We evaluated the effects of inflammatory cytokines on the gene and protein expression of lipogenic and oxidative genes in palmitate-treated primary mouse hepatocytes. Palmitate treatment significantly increased SREBP1, FAS, ACC, CPT1A, CROT and HADHB expression in primary hepatocytes. Both TNFα and IL-6 increased lipogenic gene (SREBP1, FAS and ACC) expression and decreased oxidative gene (CPT1A, CROT and HADHB) expression compared with the baseline values of palmitate-treated cells (Fig. 4a-d). Furthermore, inflammatory cytokine treatment increased the enzymatic activity of FAS and inhibited the enzymatic activity of CPT1 in palmitate-treated primary hepatocytes (Fig. 4e,f). These *in vitro* data are consistent with the results from the *in vivo* experiments.

### Chronic inflammation promotes both oxidative stress and endoplasmic reticulum (ER) stress in the livers of HFD-induced obese mice

**Figure 5:**
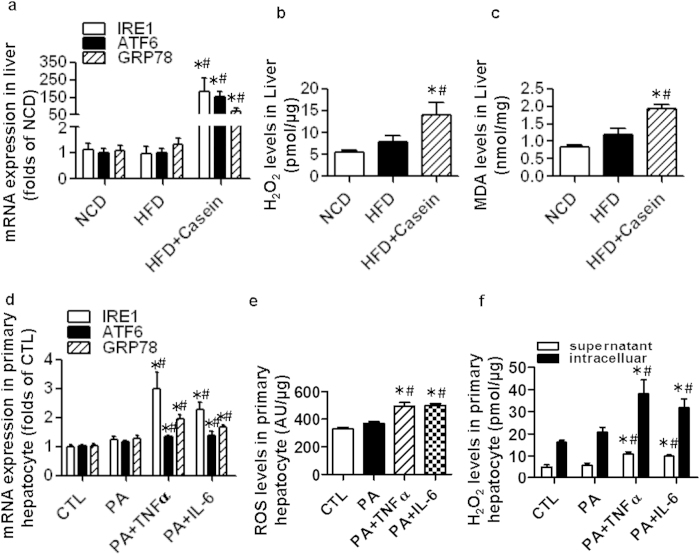
Effect of inflammatory stress on both oxidative stress and ER stress in the livers of C57BL/6J mice and in primary mouse hepatocytes. Mice were fed a normal chow diet (NCD), high-fat diet (HFD) or HFD plus casein injection (HFD + Casein) for 14 weeks. (**a**) The mRNA expression of genes associated with ER stress in the livers of C57BL/6J mice. (**b**) MDA levels in the livers of C57BL/6J mice. (**c**) H_2_O_2_ levels in the livers of C57BL/6J mice. The results are depicted as the mean ± SE (*n* = 8). *P < 0.05 versus NCD, #P < 0.05 versus HFD. Primary mouse hepatocytes were incubated in serum-free medium (control, CTL), medium containing 0.04 mM palmitate (PA), 25 ng/ml TNFα plus 0.04 mM palmitate (PA + TNFα) or 20 ng/ml IL-6 plus 0.04 mM palmitate (PA + IL-6). (**d**) Levels of ROS in primary hepatocytes. (**e**) Levels of H_2_O_2_ in the cell culture supernatant and in primary hepatocytes. The results are depicted as the mean ± SE (*n* = 6). *P < 0.05 versus CTL, #P < 0.05 versus PA.

HFD feeding alone did not upregulate the hepatic mRNA expression of ER stress markers and related genes, including inositol-requiring enzyme 1 (IRE1), activating transcription factor 6 (ATF6) and glucose-regulated protein (GRP78), all of which were significantly increased in the HFD+Casein group (Fig. 5a). Moreover, casein-injected mice had greater hydrogen peroxide (H_2_O_2_) and malondialdehyde (MDA) production in the liver compared to HFD or control mice (Fig. 5b,c). Thus, HFD feeding alone did not induce both hepatic ER and oxidative stress. These pathological conditions in the liver were aggravated in the combined presence of HFD and casein injection.

Our results also showed that TNFα and IL-6 increased the mRNA expression of IRE1, ATF6 and GRP78 in primary hepatocytes (Fig. 5d). The level of reactive oxygen species (ROS) in the cells (Fig. 5e) and the H_2_O_2_ levels in both the cell culture supernatant and the cells (Fig. 5f) were higher in cytokine-treated cells compared with palmitate-treated cells. These results suggest that inflammatory cytokines induce both ER and oxidative stress in primary mouse hepatocytes, which can lead to cell injury.

## Discussion

Numerous studies have suggested that the rate of NAFLD increases with increasing BMI^21^. However, approximately 40–50% of obese adults are “metabolically normal” (without NAFLD, diabetes mellitus or coronary heart disease)^22^. Furthermore, previous studies have indicated that systemic inflammation is strongly associated with hepatic steatosis and cardiometabolic disorders in obese individuals. The chronic systemic inflammatory state in obese patients with NAFLD is independent of BMI, percent body fat and visceral fat mass and is beyond what is explained by the presence of obesity^16^. To model these complex conditions, we studied diet-induced obese mice with and without chronic inflammation.

The central mechanism of the development of obesity is excessive caloric intake in conjunction with increased lipid ingestion. Although feeding mice a HFD is an established animal model of obesity, multiple factors, i.e., dietary components, duration of feeding and strains of mice, may affect liver phenotypes^17^,^18^. D12492, a HFD designed to induce dietary obesity, was previously reported to increase vacuolar degeneration and inflammation in the liver but usually requires a longer treatment duration (i.e., 20 weeks)^19^,^20^. Under our experimental conditions, we observed that mice with a HFD alone exhibited apparent phenotypes of obesity and dyslipidemia, without the appearance of systemic inflammation and liver steatosis. While casein injection in conjunction with a HFD had no effect on caloric intake, body weight, epididymal fat weight and serum TG or FFA levels, the injection did induce marked hepatic steatosis accompanied by elevated inflammatory cytokines in the serum and livers of HFD-fed mice. Our results suggested that the casein-induced systemic inflammation, more than HFD or obesity per se, is responsible for the intrahepatic biochemical changes observed in obesity-related NAFLD.

Simple hepatic steatosis per se might not adversely affect the outcome of NAFLD; in contrast, NASH determines the long-term prognosis of NAFLD. More than a decade ago, Day and colleagues presented an important theoretical framework termed the “two-hit hypothesis,” which suggests that after an initial hit (i.e., hepatic steatosis), the second hit (i.e., inflammation) is needed to develop NASH^23^. Various studies have investigated how inflammation acts as the so-called “second hit” in the progress of NASH^24^. However, this theory has increasingly been called into question based on the complex interactions between lipid homeostasis and inflammatory stress^25^. As a consequence, the “multiple-parallel hit” hypothesis has emerged, which suggests that hepatic steatosis in NASH may be considered a “bystander phenomenon” subsequent to inflammatory attack^26^. In the present study, we might provide additional information on the evolution of hepatic inflammation and steatosis during the progress of NAFLD. Together with previous studies by our group and others^27^,^28^, it appears that inflammatory stress is involved in the occurrence and development of NAFLD and does not occur only during the “second hit” phase.

Excessive intrahepatic TG accumulation or steatosis generally occur when there is more fatty acid synthesis and less fatty acid oxidation. Studies on obese adults with hepatic steatosis have described increased hepatic *de novo* lipogenesis accompanied with decreased fatty acid oxidation compared with matched controls without steatosis^29^,^30^. In normal subjects, *de novo* lipogenesis contributes to less than 5% of the fatty acids incorporated into secreted very low-density lipoprotein-TG (VLDL-TG). However, the rate of *de novo* fatty acid synthesis is greatly increased and accounts for approximately 20% of the fatty acids stored as intrahepatic TGs in subjects with NAFLD^31^. Genetic or experimentally induced deficiencies in mitochondrial oxidative enzymes lead to hepatic steatosis^32^,^33^; in contrast, increasing the expression or activity of hepatic enzymes involved in fatty acid oxidation reduces intrahepatic TG accumulation in rodent animal models^34^.

In our present study, we showed that *de novo* fatty acid synthesis and oxidation were both enhanced in HFD mice without casein injection. The balance between fatty acid synthesis and β-oxidation in the liver may explain the normal intrahepatic lipid content observed in the HFD-only group. However, the systemic inflammation induced by casein injection effectively increased *de novo* hepatic lipogenesis and suppressed hepatic fatty acid oxidation compared with the HFD mice not exposed to casein. The increased fatty acid input and decreased fatty acid output led to the development of liver steatosis in the HFD+Casein group. In addition, we examined the direct effect of inflammatory cytokines on fatty acid metabolism in primary mouse hepatocytes. Consistent with the *in vivo* results, treatment with TNFα or IL-6 upregulated baseline lipogenic gene expression and enzymatic activity and was accompanied by the downregulation of baseline oxidative gene expression and enzymatic activity in palmitate-treated cells.

We also observed that inflammation stimulated oxidative stress in both the livers of C57BL/6J mice and primary mouse hepatocytes, which might be attributed to cytokine-induced NADPH oxidase activation, mitochondrial dysfunction, or other enzymes related to ROS generation^35^. Both inflammatory cytokines and ROS generation could trigger ER stress^36^, as evidenced by the increase in ER stress markers (IRE1, ATF6) and the ER chaperone GRP78 in our inflamed *in vivo* and *in vitro* models. Under ER stress conditions, IRE1 can excise a 26-nucleotide fragment from the X-box binding protein-1 (XBP1) mRNA, resulting in a frameshift and consequent translation of the active transcription factor XBP1s. XBP1 can regulate fatty acid metabolism either directly and/or through other key lipogenic transcription factors, i.e., peroxisome proliferator-activated receptors (PPARs)^37^. Additionally, it has been demonstrated that ER stress can result in the generation of ROS due to Ca^2+^ release from the ER via inositol-trisphosphate receptors and enhance the inflammatory response via activating c-Jun N-terminal kinase (JNK) and IκB kinase-Nuclear factor kappa beta (IKK-NF-κB) signaling pathways^38^. Therefore, inflammation, oxidative stress and ER stress integrate to produce hepatic steatosis and liver damage^39^,^40^.

In conclusion, this study demonstrated that initial chronic inflammation is necessary to develop chronic liver disease related to steatosis under obese conditions. This mechanism might operate in obese individuals with inflammation and make them prone to develop NAFLD. Systemic inflammation could be a useful biomarker for risk assessment and a potential therapeutic target for the prevention/treatment of NAFLD in obese patients.

## Methods

### Animals

Animal care and experimental procedures were reviewed and approved by the Animal Care Committees of Chongqing Medical University on 2nd September 2011 in accordance with the National Institutes of Health Guide for the Care and Use of Laboratory Animals (NIH publication number 8023, revised 1978). For the control group, eight-week-old male C57BL6/J mice were fed a normal chow diet composed of 10% kcal fat (D12102C, Research Diets Inc., New Brunswick, NJ, USA). For the high-fat diet (HFD) group (n = 8) and HFD+Casein group (n = 8), mice were fed a diet containing 60% kcal fat (D12492; Research Diets Inc., New Brunswick, NJ, USA). In addition, the HFD+Casein mice were subcutaneously injected with 0.5 ml of 10% casein solution, while the control and HFD mice were subcutaneously injected with an equal volume of normal saline every other day. Experiments were terminated after 14 weeks of treatment.

### Cell culture

Primary hepatocytes were isolated from the livers of C57BL/6J mice using a collagenase IV perfusion method. The experimental medium was prepared with DMEM-F12 medium containing 0.2% fatty acid-free bovine serum albumin (Sigma-Aldrich Corp, St. Louis, MO, USA). Palmitate (0.04 mM), protease inhibitor cocktail, malonyl-CoA, acetyl-CoA, palmitoyl-coenzyme A (palmitoyl-CoA), 5,5’-dithio-bis-(2-nitrobenzoic acid) (DTNB) and L-carnitine were purchased from Sigma (Sigma-Aldrich Corp, St. Louis, MO, USA). TNFα (25 ng/ml) and IL-6 (20 ng/ml) were purchased from Peprotech (Peprotech, Rocky Hill, NJ, USA) and SinoBio (SinoBio Biotech, Shanghai, China), respectively.

### Measurements of serum parameters

Mice were deprived of food overnight, and serum was collected. The serum cytokine content was measured using a milliplex enzyme-linked immunosorbent assay (ELISA) assay kit (Millipore, Billerica, MA, USA). Serum FFA levels were also measured with an ELISA kit (Cusabio, Wuhan, China). Serum levels of fasting glucose, TG, AST and ALT were determined by an automatic biochemistry analyzer.

### Histological analysis

Mouse livers were collected and sequentially fixed, dehydrated, infiltrated and cut into 5-μm-thick paraffin-embedded tissue sections. Each section was routinely stained with H&E. For immunohistochemistry staining, the sections were stained using commercial kits (ZsBio, Beijing, China) following the manufacturer’s instructions. Anti-F4/80 was purchased from Biolegend (San Diego, CA, USA). The sections were also staining with Sirius red solution for collagens and the Sirius red-positive areas in 6 separated fields from different mice of each group were analyzed using Image J software. Frozen liver sections were stained with Oil Red O.

### Quantitative measurement of intrahepatic TG and FFA content

TG and FFA content in the liver was analyzed using commercial kits (Dongou, Zhejiang, China and Cusabio, Wuhan, China). The concentrations of TG and FFA were analyzed and normalized by protein concentration.

### Reverse transcription polymerase chain reaction (RT-PCR)

Total RNA was extracted from the livers of mice or primary mouse hepatocytes by RNAiso Plus reagent (Takara, Dalian, China). Then, cDNA synthesis and quantitative real-time PCR was performed with commercial kits (Takara, Dalian, China) using the Bio-Rad CFX Connect^TM^ Real-Time System (Bio-Rad, Hercules, CA, USA) according to the manufacturer’s instructions. The primer pairs used are listed in Table 2. The relative expression levels of objective messenger RNA (mRNA) were calculated as a ratio to β-actin gene expression.

### Western blot analysis

The total protein or nuclear protein from liver homogenates or cells was extracted using commercial kits (Keygen, Nanjing, China). Sample proteins were separated by sodium dodecyl sulfate polyacrylamide gel electrophoresis in a Bio-Rad Mini protean apparatus (Bio-Rad, Hercules, CA, USA) and transferred to a PVDF membrane (Millipore, Billerica, MA, USA). The membranes were then blocked and incubated with primary antibodies (anti-SREBP-1, anti-ACC, anti-FAS, anti-CPT1A, anti-HADHB, anti-CROT, Santa Cruz Biotechnology, Dallas, Texas, USA) followed by incubation with a horseradish peroxidase-labeled secondary antibody (Santa Cruz Biotechnology, Dallas, Texas, USA). Densitometric analysis was performed directly from the blotted membrane using the Fusion FX5 imaging system (Vilber Lourmat, Marne, France).

### ROS, H_2_O_2_ and MDA assays

The hepatic ROS and H_2_O_2_ content was measured using the ROS and hydrogen peroxide assay Kit (Beyotime, China) according to the manufacturer’s instructions and normalized by protein concentration. We also measured the hepatic MDA content using a commercial kit (Jiancheng, Nanjing, China) and normalized by total liver protein.

### FAS activity assay

Cells were harvested, and the homogenates was centrifuged at 12,000 g for 5 min. FAS activity was measured in the supernatants spectrophotometrically by monitoring the oxidation of NADPH at 340 nm as previously described^41^. The protein concentration of the cleared supernatant was determined by the Lowry method. Enzymatic activity was defined as μM NADPH oxidized per minute per milligram of protein.

### CPT1 activity assay

Cells were harvested, and the cell homogenates were centrifuged at 12,000 x *g* for 5 min. CPT1 activity was assayed in the supernatants spectrophotometrically by following the release of CoA-SH from palmitoyl-CoA using the general thiol reagent DTNB as previously described^42^. The protein content of the cleared supernatants was determined using the Bradford method. CPT1 activity was defined as nM CoA-SH released/min/mg protein.

### Statistical analysis

The data are expressed as the mean ± SE. All experimental data were evaluated for statistical significance using a one-way analysis of variance followed by a Tukey-test. Differences were considered significant at *P* values less than 0.05.

## Author Contributions

All authors have contributed to and agreed on the content of this paper. L.Z., S.Z., H.Y.Q , Y.X.X., Q.L., P.Y., and Z.N.C. performed the research; L.Z. and S.Z. analyzed the data; Z.S., L.Z., H.Y.Q. and Y.X.C. wrote the paper; Y.X.C. designed the research study; Z.V., J.F.M. and X.Z.R. edited and revised manuscript; Y.X.C. and X.Z.R. interpreted results of experiments; Y.X.C. approved final version of manuscript. All authors reviewed the manuscript.

## Additional Information

**How to cite this article**: Zhao, L. *et al*. Chronic inflammation aggravates metabolic disorders of hepatic fatty acids in high-fat diet-induced obese mice. *Sci. Rep.* **5**, 10222; doi: 10.1038/srep10222 (2015).

## Figures and Tables

**Table 1:** Characteristics of mice after the 14-week experimental period

	**NCD**	**HFD**	**HFD + Casein**
Weight gain from 8 to 22 week (g)	4.31 ± 0.59	9.13 ± 0.62*	9.29 ± 0.60*
Body weight (g)	22.1 ± 0.49	29.56 ± 0.85*	29.18 ± 0.28*
Total calorie intake (kcal/d)	8.48 ± 0.72	12.61 ± 2.08*	12.76 ± 2.45*
Liver weight (g)	0.91 ± 0.01	0.89 ± 0.05	1.06 ± 0.05* #
Epididymal fat weight (g)	0.33 ± 0.03	0.94 ± 0.15*	0.91 ± 0.03*
Serum TG (mg/dl)	33.23 ± 5.85	52.45 ± 4.64*	42.09 ± 5.12*
Serum FFA (μg/ml)	0.59 ± 0.10	0.94 ± 0.05*	1.091 ± 0.11*
Serum fasting glucose (mmol/L)	5.94 ± 0.35	5.550 ± 0.49	8.21 ± 1.07* #
Values are the mean ± SE (n = 8).			

^*^P < 0.05 versus NCD.

^#^P < 0.05 versus HFD.

**Table 2:** Characteristics of mice after the 14-week experimental period.

**Genes**		**Mouse primers**
β-actin	Forward	5′-CGATGCCCTGAGGCTCTTT-3’
NM_007393.3	Reverse	5′-TGGATGCCACAGGATTCCAT-3′
SREBP1	Forward	5′-GCCCACAATGCCATTGAGA-3'
NM_011480.3	Reverse	5′-CAGGTCTTTGAGCTCCACAATCT-3'
FAS	Forward	5′-CCTGGATAGCATTCCGAACCT-3'
NM_007988.3	Reverse	5′-AGCACATCTCGAAGGCTACACA-3'
ACC1	Forward	5′-CGCTCAGGTCACCAAAAAGAAT-3'
NM_133360.2	Reverse	5′-GTCCCGGCCACATAACTGAT-3'
HADHB	Forward	5′-TGGCTGTCGGCTGGTCAT-3′
NM_145558.1	Reverse	5′-AGCATACTGGCCTCCATCCTT-3'
CPT1	Forward	5′-GAACCCCAACATCCCCAAAC-3′
NM_013495.2	Reverse	5′-CCTGGCATTCTCCTGGAATG-3'
CROT	Forward	5′-CTTTTACCACGGCCGAACA-3′
NM_023733.3	Reverse	5′-CCTGACGGCCTCCACTGTA-3'
IRE1	Forward	5′-GGCTACTCATTGGATACCACGAA-3′
NM_012016.2	Reverse	5′- CCCGCAGCATGGTTGTG-3′
ATF-6	Forward	5′-CCCTTATGCCACTGGCAAA-3′
NM_001081304.1	Reverse	5′- GGGCGCAGGCTGTATGC-3′
GRP78	Forward	5′-AGCCATCCCGTGGCATAA-3′
NM_001163434	Reverse	5′- GGACAGCGGCACCATAGG-3′
